# Mixed methods in pre-hospital research: understanding complex clinical problems

**DOI:** 10.29045/14784726.2020.12.5.3.44

**Published:** 2020-12-01

**Authors:** Gregory Adam Whitley, Scott Munro, Pippa Hemingway, Graham Richard Law, Aloysius Niroshan Siriwardena, Debbie Cooke, Tom Quinn

**Affiliations:** University of Lincoln ORCID iD: https://orcid.org/0000-0003-2586-6815; University of Surrey; South East Coast Ambulance Service NHS Foundation Trust ORCID iD: https://orcid.org/0000-0002-0228-4102; University of Nottingham ORCID iD: https://orcid.org/0000-0003-1944-8166; University of Lincoln ORCID iD: https://orcid.org/0000-0001-7904-0264; University of Lincoln ORCID iD: https://orcid.org/0000-0003-2484-8201; University of Surrey ORCID iD: https://orcid.org/0000-0003-1944-7905; Kingston University & St George’s, University of London ORCID iD: https://orcid.org/0000-0002-5116-0034

**Keywords:** emergency medical services, mixed methods, research design

## Abstract

Healthcare is becoming increasingly complex. The pre-hospital setting is no exception, especially when considering the unpredictable environment. To address complex clinical problems and improve quality of care for patients, researchers need to use innovative methods to create the necessary depth and breadth of knowledge. Quantitative approaches such as randomised controlled trials and observational (e.g. cross-sectional, case control, cohort) methods, along with qualitative approaches including interviews, focus groups and ethnography, have traditionally been used independently to gain understanding of clinical problems and how to address these. Both approaches, however, have drawbacks: quantitative methods focus on objective, numerical data and provide limited understanding of context, whereas qualitative methods explore more subjective aspects and provide perspective, but can be harder to demonstrate rigour. We argue that mixed methods research, where quantitative and qualitative methods are integrated, is an ideal solution to comprehensively understand complex clinical problems in the pre-hospital setting.

The aim of this article is to discuss mixed methods in the field of pre-hospital research, highlight its strengths and limitations and provide examples. This article is tailored to clinicians and early career researchers and covers the basic aspects of mixed methods research. We conclude that mixed methods is a useful research design to help develop our understanding of complex clinical problems in the pre-hospital setting.

## Background

Evidence based medicine (EBM) involves the ‘conscientious, explicit and judicious use of current best evidence’ (Sackett et al., 1996, p. 71). EBM has been accepted as the ‘gold standard’ for pre-hospital healthcare development and improvement, and results from the integration of best evidence, individual clinical expertise, patient preferences and values (Swanson et al., 2010).

Evidence informing the treatment and management of patients in the pre-hospital environment has primarily consisted of quantitative research (McManamny et al., 2014), including randomised controlled trials (RCTs) and cohort studies. The volume of qualitative research has been steadily increasing in recent years, including phenomenological, ethnographic and grounded theory approaches (Green and Thorogood, 2018). Individual quantitative studies such as RCTs aim to determine the efficacy or effectiveness of treatments and interventions (Law and Pascoe, 2013). RCTs are designed to exclude the possibility of confounding variables accounting for observed results. Their aim is to determine causation where conclusions are drawn based on appropriate numbers of patients. With appropriate sample selection, the results should be generalisable. However, quantitative research is objective and provides a limited understanding of context (Creswell, 2014). As pre-hospital healthcare research involves people, qualitative methodologies are useful to understand lay and professional views, attitudes, beliefs and behavioural intentions (Pope and Mays, 1995), as well as experiences and cultures (Al-Busaidi, 2008). A limitation of individual qualitative research is the subjective nature of the findings (Creswell, 2014), which can be difficult to demonstrate rigour (Cypress, 2017).

Considering the inherent disadvantages of quantitative and qualitative research, coupled with increasing healthcare complexity (Plsek and Greenhalgh, 2001), we argue for the use of mixed methods research. This is particularly helpful in the pre-hospital setting due to the unpredictable environment (Abelsson and Lindwall, 2012), where mixed methods are an ideal solution to the difficulty faced when attempting to fully understand complex clinical problems. The aim of this article is to discuss mixed methods in the field of pre-hospital research, highlight its strengths and limitations and provide examples from our own research.

## What is mixed methods research?

Though there is no formally established or universally agreed definition of mixed methods research (Johnson et al., 2007), Creswell (2014) defines it as the integration of quantitative (numerical) *and* qualitative (non-numerical) data within one overall study. The collective strength of combining both types of data provides a better understanding of the research problem than can be achieved with either form of data alone (Creswell, 2014).

The integration of quantitative and qualitative data from two or more studies is considered essential to mixed methods research (Creswell, 2014; O’Cathain et al., 2010). Integration can be achieved at the design, methods, interpretation and reporting level. This is achieved via connecting, building, transforming (Fetters et al., 2013), following a thread, triangulation (O’Cathain et al., 2010) or using a joint display (Guetterman et al., 2015), among other techniques. See Table 1 for a description of these methods. Without integration, a study involving quantitative and qualitative data has been termed ‘multi-methods’ (Creswell, 2014).

**Table 1.** Methods of integration.

Level	Method	Description
**Design**	Sequential explanatory Sequential exploratory Convergent	Data are inherently integrated in these designs, as they are *explained* (sequential explanatory), *tested* (sequential exploratory) or *merged* (convergent).
**Methods**	Connecting Building Following a thread	When the findings from one study inform the sampling of the other. When the findings from one study inform the data collection approach of the other. When a question or theme from one study is followed across to the other study to elicit deeper understanding.
**Interpretation and reporting**	Triangulation Data transformation Joint display	Assessing the level of agreement, complementarity^a^ and contradiction between both sets of findings. Transforming one type of data into the other, followed by combining the data. Visually bringing together quantitative and qualitative findings into a figure or table to facilitate the generation of meta-inferences.^b^

Adapted from Fetters et al. (2013), Guetterman et al. (2015) and O’Cathain et al. (2010).

^a^ Quantitative and qualitative data may address different aspects of a phenomenon and therefore may not be able to confirm or refute each other; instead they may offer complementary information which can help build a more comprehensive understanding of the problem.

^b^ ‘[A] conclusion generated by integrating the inferences obtained from the qualitative and quantitative strands of a mixed methods study’ (adapted from Teddlie and Tashakkori, 2009, p. 338).

The aim of mixed methods research is to create depth and breadth of understanding (Johnson et al., 2007) that is considered more than, or beyond, the sum of its parts (Teddlie and Tashakkori, 2009). Such conclusions are termed ‘meta-inferences’, defined as: ‘a conclusion generated by integrating the inferences obtained from the qualitative and quantitative strands of a mixed methods study’ (adapted from Teddlie and Tashakkori, 2009, p. 338).

## Types of mixed methods research

For the purpose of this article, we have only discussed the three main basic types of mixed methods design described by Creswell (2014): sequential explanatory, sequential exploratory and convergent. More advanced designs include the embedded (or intervention) design, where qualitative research is incorporated into quantitative experimental research. An example of this is a process evaluation of an RCT, to better understand the patient’s experience of the intervention or of the trial itself (OʼCathain et al., 2013), including examination of how the intervention is working and how, if it is successful, it may be sustained or spread (Moore, et al., 2015).

### Sequential design

**Figure 1:**
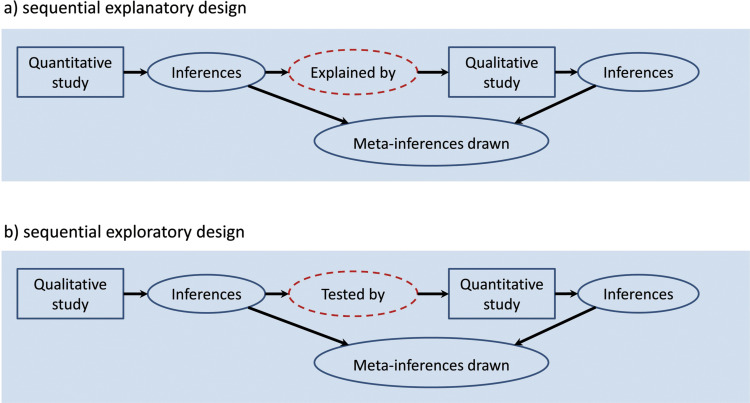
Figure 1. Diagram of procedures for mixed methods sequential designs. Adapted from Creswell (2014); Teddlie and Tashakkori (2009).

The sequential design is the most popular mixed methods approach in pre-hospital research (McManamny et al., 2014). It can either be explanatory: quantitative findings are *explained* by qualitative data; or exploratory: qualitative findings are used to generate hypotheses that are *tested* by quantitative methods (Schoonenboom & Johnson, 2017). Figure 1 shows a diagram of procedures for both types of sequential design.

The sequential explanatory design (Figure 1a) is often utilised by researchers who have a background in quantitative research (Creswell, 2014), due to the familiarity of the initial statistical phase. The conclusions, or ‘inferences’, generated from the initial quantitative study are observational in nature and offer little depth of understanding or explanation, hence the need for a second qualitative phase to *explain* the findings.

The sequential exploratory design is often used when little is known about a topic, for example due to an understudied population (Creswell, 2014). The hypotheses and theories generated from the initial qualitative work can then be *tested* quantitatively (see Figure 1b).

One of the major drawbacks of the sequential designs is the time taken to perform the overall study: they must be performed sequentially, as the findings from the initial study are needed to inform the second study. This drawback, however, is also a benefit; integration occurs when the initial study informs the second study in the form of ‘connecting’ (when the findings from one study inform the sampling of the other) and ‘building’ (when the findings from one study inform the data collection approach of the other) (Fetters et al., 2013).

### Convergent design

**Figure 2:**
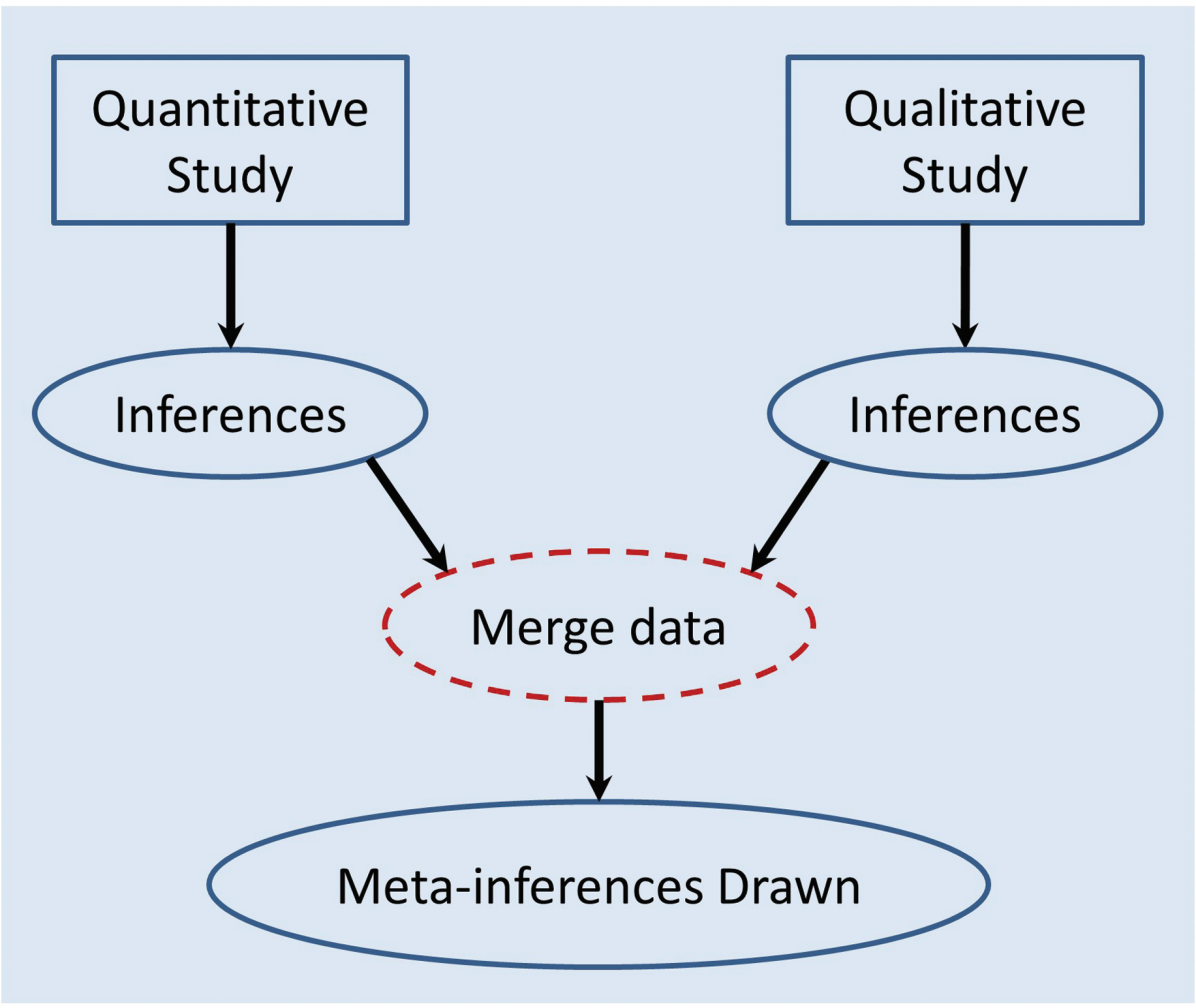
Figure 2. Diagram of procedures for mixed methods convergent design. Adapted from Creswell (2014); Teddlie and Tashakkori (2009)

The convergent design (see Figure 2) involves the separate and often simultaneous collection and analysis of quantitative and qualitative data (Creswell, 2014). The separate findings are then merged through techniques such as triangulation, which is the ‘process of studying a problem using different methods to gain a more complete picture’ (O’Cathain et al., 2010, p. 1147) by assessing the level of agreement, complementarity and contradiction between both sets of findings. Triangulation may identify contradiction in the data; this is not a failure of the research but an important part of the process, as discrepancy may lead to a better understanding of the research question (O’Cathain et al., 2010).

Meta-inferences produced from triangulation techniques may be illustrated within a joint display, providing visual structure and facilitating the process of integration (Guetterman et al., 2015). Another method of merging data is through transformation: when qualitative data is transformed into quantitative form, or vice versa (Fetters et al., 2013).

A major benefit of the convergent design is that quantitative and qualitative data can be collected at the same time, significantly reducing the time taken compared to sequential designs (Creswell, 2014). However, the process of analysis is arguably more challenging, especially when performing data transformation (Fetters et al., 2013).

## Strengths of mixed methods research

Findings from mixed methods studies are considered more than the sum of their parts (Teddlie and Tashakkori, 2009). Integrating statistical analysis with a rich understanding of concepts generated through qualitative methodologies provides a better understanding of the research problem (Creswell, 2014) than performing two separate studies in isolation. This allows for a deeper understanding of complex clinical problems from well-designed and conducted mixed methods studies than can be achieved with conventional quantitative or qualitative approaches alone.

Mixed methods enables researchers to ask exploratory and confirmatory questions at the same time, thus generating and verifying theories within the same study (Teddlie and Tashakkori, 2009) through the process of ‘following a thread’ (O’Cathain et al., 2010). This unique ability allows questions to be asked and answered in a rapid iterative fashion. From the personal experiences of the authors, mixed methods research allows many more questions to be answered than can be achieved with a single research method. Study designs that are reliant exclusively on a quantitative or qualitative approach often lead to many more questions, which take time to answer as new studies must be set up to answer them.

## Limitations of mixed methods research

A major limitation of mixed methods is the increased time taken to complete the overall study, particularly with sequential designs (Hansen et al., 2016). This must be considered early in the development phase because data collection, analysis and interpretation of two or more studies take a significant amount of time.

Individual researchers may not have the skill set to undertake a mixed methods study, and therefore additional researchers may be required to assist with the project (Hansen et al., 2016).

Due to the recent history of mixed methods research, the quality, validity and reliability of the meta-inferences generated can be difficult to judge (Teddlie and Tashakkori, 2009). It has been argued that design quality and interpretive rigour should be assessed to determine inference quality, as set out by Teddlie and Tashakkori (2009: p. 301) within their ‘integrative framework for inference quality’. The good reporting of a mixed methods study (GRAMMS) criteria has been proposed as a mixed methods reporting guideline (OʼCathain et al., 2008).

Another limitation is the publication process: many journals limit the word count, making it difficult to publish a full mixed methods study within the word limit without losing necessary detail. A pragmatic approach is to either append data as a supplementary file or publish the studies separately, although care must be taken not to lose the depth of integration. OʼCathain et al. (2007) argued that separate publication of studies within a mixed methods approach *may* produce a result that is ‘the sum of its parts’ rather than ‘more than the sum of its parts’, potentially negating the inherent strength of performing mixed methods research. Detailed explanation of the level of integration achieved and comprehensive discussion of the meta-inferences are required if a model of separate publication is to be adopted as the dissemination strategy.

A further limitation is the philosophical argument that quantitative and qualitative paradigms or ‘worldviews’ are incommensurable (Tashakkori and Teddlie, 2010), leading some to think that the methodology is inherently flawed. Tashakkori and Teddlie (2010) offer solutions to this argument, along with a comprehensive discussion of contemporary issues regarding mixed methods research which is beyond the scope of this article, though we recommend this as further reading.

To demonstrate how mixed methods can be practically applied to pre-hospital research, we have provided details of two research studies; see examples 1 and 2.

## Example 1: Pre-hospital child pain management

Pre-hospital child pain management is an extremely complex phenomenon: the illness or injury must be considered along with the child’s perception of pain (influenced by many factors), the ambulance clinician’s ability to assess and manage the pain, the role of the parents and the theory of pain (Whitley et al., 2019). Child pain management in the ambulance service is considered poor (Samuel et al., 2015). Before improvements can be made, the problem must be fully understood. It was unlikely that individual quantitative or qualitative studies would create findings of sufficient depth and breadth to fully understand the problem, therefore a mixed methods approach was utilised.

We were interested to know which children were likely to achieve effective pain management (defined as the abolition or reduction of pain ≥2 out of 10) and to explore potential reasons for any disparity. The sequential explanatory design was adopted (Figure 1a), and predictors of effective pain management were identified using electronic data from completed clinical records; this formed the initial quantitative study (Whitley, Hemingway, Law, Wilson et al., 2020).

**Figure 3:**
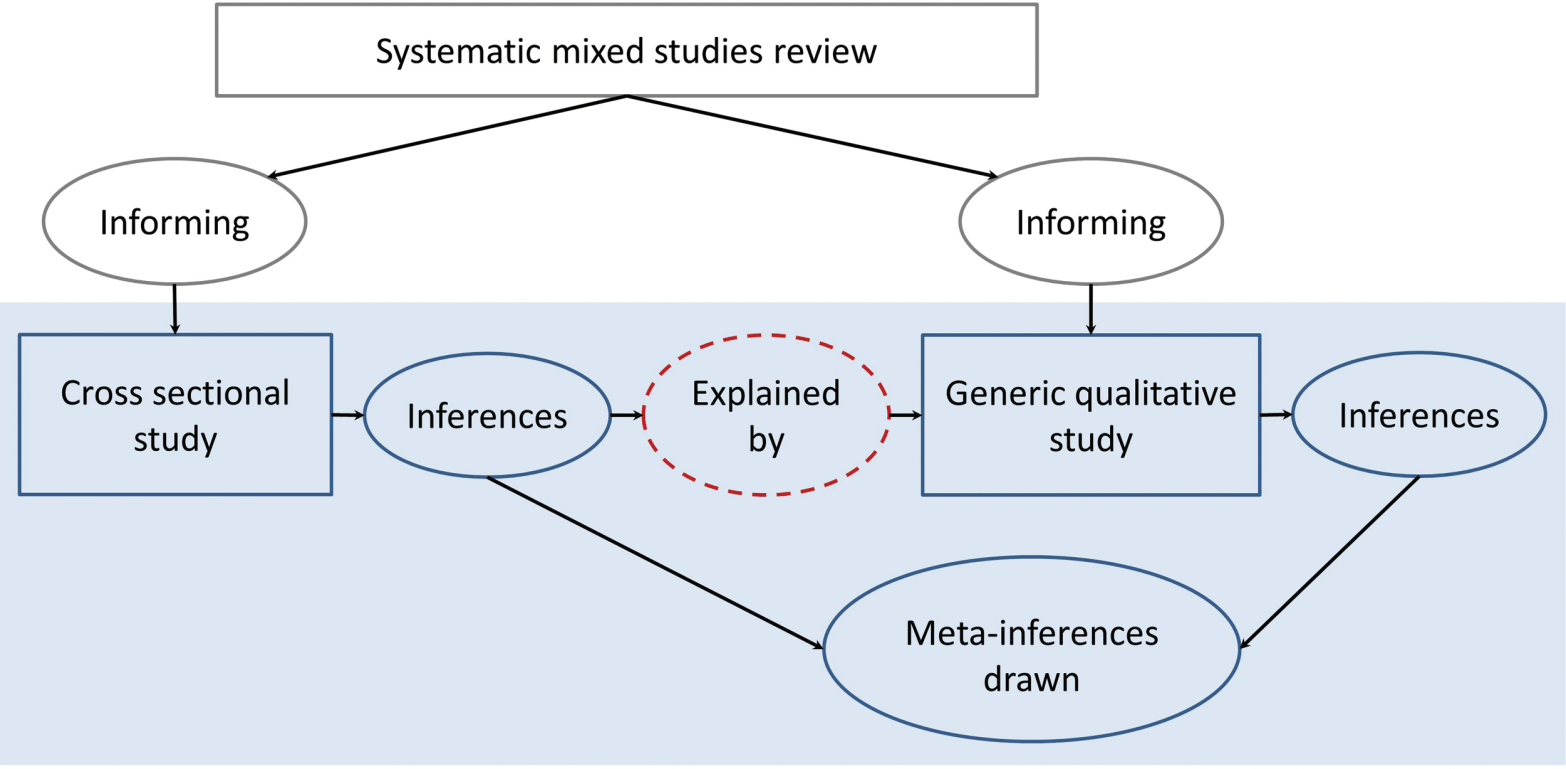
Figure 3. Diagram of procedures for the pre-hospital child pain management study.

A qualitative study was then used to *explain* the predictors of effective pain management; see Figure 3 for the diagram of procedures.

Figure 3 shows that the mixed methods study was informed by a systematic mixed studies review (Whitley, Hemingway, Law, Jones, et al., 2020): previously identified predictors of effective pain management in children were included in our multivariable logistic regression analysis and previously identified barriers and facilitators were explored further during the qualitative study. The objectives of the qualitative study were: a) to explain the identified predictors of effective pain management (completing the mixed methods sequential explanatory study), b) to identify barriers and facilitators and c) to explore ways to improve pain management. We therefore used the qualitative study for more than explanatory purposes. Considering participants were already in a face-to-face interview with their mind focused on the specific topic of pre-hospital child pain management, it was a pragmatic choice to seek more than explanation, but to also explore barriers, facilitators and potential improvements.

One of the key benefits of using a mixed methods approach was that it allowed us to ‘follow a thread’ (O’Cathain et al., 2010) to help explain findings that could not be explained comprehensively using qualitative techniques. For example, our quantitative study found that children living in more deprived areas were less likely to achieve effective pain management (Whitley, Hemingway, Law, Jones et al., 2020). When asked at interview, participants gave a broad variety of reasons for this finding, none of which fully explained the difference. We re-examined the statistical data using some of the theories generated from the interviews and were able to strengthen some of the explanations with quantitative data. This developed a more comprehensive explanation for the disparity. Without the mixed methods approach, this depth of knowledge may not have been gained.

We chose a method of separate publication for pragmatic reasons, and published our cross-sectional study first (Whitley, Hemingway, Law, Wilson et al., 2020). We aim to publish our explanation of predictors as a separate study which will include a detailed explanation of the integration achieved along with a discussion of the meta-inferences.

## Example 2: Pre-hospital stroke care

Stroke is a leading cause of death and disability across the globe (Gorelick, 2019; Johnson, et al., 2019). Emergency medical services (EMS) play a vital role in the recognition, management and transportation of stroke patients to hospital (Munro et al., 2018). Prior to 2019, the UK national clinical practice guidelines, set out by the Joint Royal Colleges Liaison Committee (JRCALC), recommended that EMS staff consider recording a pre-hospital 12-lead electrocardiogram (PHECG) for stroke patients, providing this did not cause significant delay (Joint Royal Colleges Ambulance Liaison Committee, Association of Ambulance Chief Executives, 2013, 2016). This recommendation was based on expert opinion, rather than robust evidence. A systematic review (Munro et al., 2018) found no studies undertaken in the pre-hospital environment investigating the use of 12-lead ECGs in acute stroke patients at the time. This led to the generation of two research questions:

1. Are there differences in functional outcome, mortality rates and processes of care in pre-hospital acute stroke patients with and without PHECG recorded?
2. What are the views, practice, attitudes towards and perceived value of recording a PHECG from the perspective of different stakeholders involved in care of this patient group?

**Figure 4:**
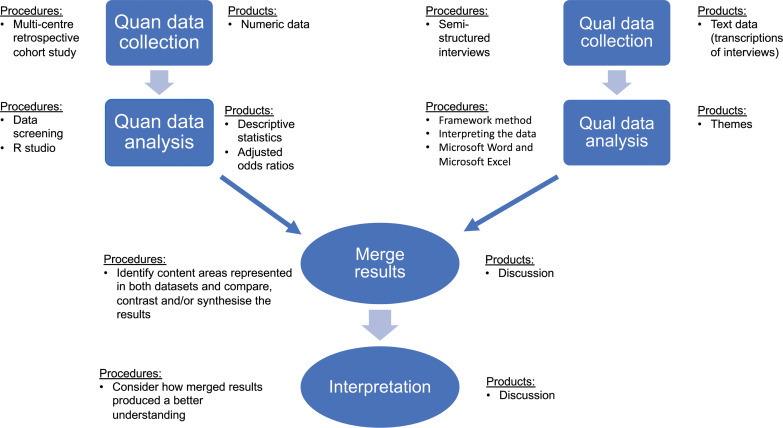
Figure 4. Diagram of procedures for the pre-hospital stroke care study.

In order to address these questions and gain a more complete understanding of the use and impact of PHECG, a mixed methods approach within a critical realist paradigm (Bhaskar, 1975, 2009, 2014) was used. The convergent design (Figure 2) was used, incorporating a quantitative linked retrospective cohort study (Study 1) and a cross-sectional qualitative interview study (Study 2); see Figure 4 for the diagram of procedures.

A mixed methods design was adopted to develop a more comprehensive understanding of the use and impact of the PHECG in acute stroke patients, which could not be achieved by adopting one method alone (Bryman, 2006). The authors believed that the phenomenon of recording PHECG for stroke patients was too complex to be fully captured by quantitative enquiry alone, and a qualitative exploration was needed to provide additional detail and understanding (Ritchie, 2003).

Study 1 was a multicentre retrospective cohort study, which linked data collected from the participating EMS trusts’ patient clinical records (PCRs) with routinely collected data from three hospitals with hyperacute stroke units. Ordinal and logistic regression analyses were used to investigate the association between patients who received a PHECG and functional outcome at discharge from hospital (measured using the modified Rankin Scale), hospital mortality rate, pre-hospital interval time, rate of thrombolysis and door-to-scan and door-to-needle time.

While the quantitative methods of Study 1 could address ‘what’, ‘who’ and ‘when’ questions (Crabtree and Miller, 1999; Silverman, 2000), they were not able to adequately answer ‘how’ or ‘why’ questions which help give a more complete picture of the process of EMS stroke patient management (Denzin and Lincoln, 2011; Silverman, 2000). Study 2 was a cross-sectional qualitative interview study, exploring the views, practice, attitudes towards and perceived value of EMS undertaking PHECGs from the stakeholders involved in the care of acute stroke patients. The PHECG decision-making process of paramedics was also explored using the cognitive continuum theory (Hamm, 1988; Standing, 2008). A purposeful sample of 14 paramedics, two emergency department nurses, three stroke nurses and three stroke physicians was recruited. Data were collected via semi-structured interviews. Themes were generated using the Framework Analysis method (Ritchie and Lewis, 2003).

Both studies were undertaken concurrently, and the results were merged and integrated in a final discussion chapter. Once the quantitative and qualitative results had been identified, common concepts across both sets of findings were identified using triangulation techniques (O’Cathain et al., 2010). Merging both sets of results created a deeper understanding of the use and impact of PHECGs for acute stroke patients in the pre-hospital setting; this was presented as a narrative discussion. The findings from Study 2 have helped to contextualise and complement the findings from Study 1, providing a deeper, different and augmented level of understanding of the phenomenon, aiding in the guidance of generating recommendations for future practice and research.

## Conclusion

Mixed methods is a useful approach to help understand complex clinical problems in the pre-hospital setting. Researchers should be aware of the strengths and limitations before embarking on a mixed methods project. We have provided two examples of how mixed methods research has been used in the pre-hospital setting and hope these provide helpful illustrations of these approaches for clinicians and early career academics who wish to undertake mixed methods research.

## Author contributions

All authors made substantial contributions to the conception and design of the mixed methods studies provided as examples in this article. GAW and SM drafted the manuscript and all authors contributed to its revision and agreed on the final version. GAW acts as the guarantor for this article.

## Conflict of interest

None declared.

## Ethics

Not required.

## Funding

This study is funded by the National Institute for Health Research (NIHR) Applied Research Collaboration East Midlands (ARC EM). The views expressed are those of the author(s) and not necessarily those of the NIHR or the Department of Health and Social Care. This work was supported by a School of Health Sciences PhD studentship funded jointly by the University of Surrey and the South East Coast Ambulance Service NHS Foundation Trust, UK.
